# Removal of Both Ovaries—Effect

**Published:** 1874-09

**Authors:** C. G. Wheelhouse


Removal of Both Ovaries.—*By Mr. C. G. Wheelhouse.*—It is three years ago this month since the operation was performed, and I have watched the case ever since with the greatest anxiety. There has never been any attempt at menstruation, nor has any vicarious discharge ever taken the place of the natural one; but, beyond this, I see no change of any kind. The general health is now as perfect as it ever was, and, so far from any uncomfortable symptoms of masculinity having occurred, the voice remains as soft, the bust as full, and the whole aspect and demeanor are as perfectly feminine as any other young lady of my acquaintance. *The Medical News and Library—July.*

